# Intracellular Lipid Accumulation and Mitochondrial Dysfunction Accompanies Endoplasmic Reticulum Stress Caused by Loss of the Co-chaperone *DNAJC3*

**DOI:** 10.3389/fcell.2021.710247

**Published:** 2021-10-06

**Authors:** Matthew J. Jennings, Denisa Hathazi, Chi D. L. Nguyen, Benjamin Munro, Ute Münchberg, Robert Ahrends, Annette Schenck, Ilse Eidhof, Erik Freier, Matthis Synofzik, Rita Horvath, Andreas Roos

**Affiliations:** ^1^Department of Clinical Neuroscience, University of Cambridge, Cambridge, United Kingdom; ^2^Leibniz-Institut für Analytische Wissenschaften – ISAS – e.V., Dortmund, Germany; ^3^Department of Human Genetics, Donders Institute for Brain, Cognition and Behavior, Radboud University Medical Center, Nijmegen, Netherlands; ^4^Department of Neurodegenerative Diseases, Hertie Institute for Clinical Brain Research, University of Tübingen, Tübingen, Germany; ^5^German Centre for Neurodegenerative Diseases (DZNE), Tübingen, Germany; ^6^Department of Pediatric Neurology, Developmental Neurology and Social Pediatrics, Children’s Hospital University of Essen, Essen, Germany

**Keywords:** proteomics, cholesterol-stress, mitochondria, *DNAJC3*, unfolded protein response (UPR)

## Abstract

Recessive mutations in *DNAJC3*, an endoplasmic reticulum (ER)-resident BiP co-chaperone, have been identified in patients with multisystemic neurodegeneration and diabetes mellitus. To further unravel these pathomechanisms, we employed a non-biased proteomic approach and identified dysregulation of several key cellular pathways, suggesting a pathophysiological interplay of perturbed lipid metabolism, mitochondrial bioenergetics, ER-Golgi function, and amyloid-beta processing. Further functional investigations in fibroblasts of patients with *DNAJC3* mutations detected cellular accumulation of lipids and an increased sensitivity to cholesterol stress, which led to activation of the unfolded protein response (UPR), alterations of the ER-Golgi machinery, and a defect of amyloid precursor protein. In line with the results of previous studies, we describe here alterations in mitochondrial morphology and function, as a major contributor to the *DNAJC3* pathophysiology. Hence, we propose that the loss of *DNAJC3* affects lipid/cholesterol homeostasis, leading to UPR activation, β-amyloid accumulation, and impairment of mitochondrial oxidative phosphorylation.

## Introduction

DnaJ Heat Shock Protein Family (Hsp40) Member C3 (DNAJC3, p58^*IPK*^) is an endoplasmic reticulum (ER)-resident co-chaperone of BiP (GRP78, HSPA5), which transiently binds to a broad range of newly synthesized proteins present in the ER to impede the misfolding of susceptible domains. In its “rested” state, DNAJC3 is localized to the ER lumen. The N-terminus of the protein is able to directly bind to hydrophobic regions of misfolded proteins present in the ER (Rutkowski et al., 2007). Once the misfolded protein is bound to the N-terminus of DNACJ3, further ATP-dependent interaction *via* the J-domain with BiP supports the refolding of the misfolded protein (Oyadomari et al., 2006). Additionally, activated DNAJC3 is thought to downregulate the signaling of the unfolded protein response (UPR) effector PERK [protein kinase R (PKR)-like endoplasmic reticulum kinase], presumably by interaction with its cytoplasmic domain (Yan et al., 2002). Dimerization of PERK *via* the phosphorylation of eukaryotic initiation factor 2 alpha (eIF2-α) reduces protein synthesis. Therefore, inhibition of PERK dimerization by DNAJC3 increases protein synthesis and, during periods of sustained UPR signaling, assists in returning the ER to normal homeostasis. Dysregulation of this process may therefore have toxic effects on cellular protein homeostasis. DNAJC3 affects ER maintenance and apoptosis, particularly under conditions of cellular stress, and it has been highlighted as a protective factor in retinal neurons (Boriushkin et al., 2015).

Mutations in components of the UPR frequently cause neurodegenerative diseases. Expression of mutant BiP lacking the carboxyl terminal in a mouse model results in neurodegeneration and severe muscle weakness, associated with aggregation of proteins in the spinal cord alongside upregulation of UPR-related proteins such as GRP94, calnexin and DDIT3 (Jin et al., 2014). Similarly, transgenic mice expressing just 50% of the wild-type BiP exhibit disruption of neural development, defects of cortical neurons, and cerebellar abnormalities, while homozygous loss-of-function mutations result in intrauterine death in mice (Luo et al., 2006). Biallelic mutations in the BiP co-chaperone *SIL1* cause Marinesco–Sjögren syndrome (MSS) in humans, a condition characterized by cataracts, early-onset cerebellar ataxia, cognitive deficits, short stature, and progressive vacuolar myopathy (Anttonen et al., 2005; Senderek et al., 2005; Krieger et al., 2013). The MSS-mouse-model (*woozy* mouse) lacks a functional *Sil1* gene and replicates the human phenotype (Buchkremer et al., 2016). Pathological examination of the *woozy* mouse reveals the loss of Purkinje cells associated with the accumulation of protein aggregates (Zhao et al., 2010), a process found to be accelerated by additional suppression of GRP170, another BiP interacting protein. This process is partially ameliorated by loss of Dnajc3 (Zhao et al., 2010). Furthermore, mutations in the BiP co-chaperone DNAJB2 have been found to be causative for distal hereditary motor neuropathy, accompanied by an increased rate of SOD1 inclusion formation in a neural cell model (Blumen et al., 2012). These studies suggest a causal relationship between dysfunction of protein processing and ER stress and the manifestation of neurological diseases.

Autosomal recessive mutations in *DNAJC3* have been first reported to cause a neurological disorder in two Turkish families, in which individuals presented with ataxia, upper-motor neuron signs, demyelinating neuropathy, neuronal hearing loss, and cerebral atrophy, complicated with early-onset type 2 diabetes mellitus (MODY, OMIM #616192) (Synofzik et al., 2014) and hypothyroidism in a distant relative of the originally reported family (Bublitz et al., 2017). Moreover, isolated type 2 diabetes mellitus has been reported in association with the heterozygous c.712C > A (p.His238Asn) *DNAJC3* mutation (Kulanuwat et al., 2018); however, the same mutation was also observed in two non-diabetic individuals in the study and has been seen at a low frequency in the ExAC database, questioning the role of *DNAJC3* as a dominant type 2 diabetes-associated gene. The p.His238Asn mutation caused minor decrease in DNAJC3 expression, in contrast to the apparent absence seen in the other reported cases. Moreover, two *DNAJC3* patients presenting with juvenile-onset diabetes, short stature, hypothyroidism, neurodegeneration, facial dysmorphism, hypoacusis, microcephaly, and skeletal bone deformities were described (Lytrivi et al., 2021). Symptoms resulting from mutations in *DNAJC3* are partially overlapping with phenotypes caused by mutations in *DNAJB2* and *SIL1*, but with the addition of diabetes (Bublitz et al., 2017).

The mechanisms underlying the pathophysiology of mutations in other BiP co-chaperones such as SIL1 have been extensively explored (Roos et al., 2014, 2016; Ichhaporia et al., 2015; Kollipara et al., 2017; Phan et al., 2019), while in contrast, there is little known about the precise cellular mechanisms underlying the pathogenicity of *DNAJC3* mutations. Studies of primary patient-derived fibroblasts revealed loss of the DNAJC3 protein and a structurally normal ER, and upon ER stress induction, only minor changes were observed in protein secretion and Ca^2+^ leakage (Synofzik et al., 2014). Studies on DNAJC3-silenced rat and human β cells did not affect insulin content and secretion but sensitized cells to ER stress, triggering mitochondrial apoptosis (Lytrivi et al., 2021). To further unravel this pathophysiological route here, we focused on elucidating the pathomechanism of DNAJC3-related pathology by performing proteomics in patient-derived primary fibroblasts, a suitable model to study the etiology of rare neurological diseases (Hentschel et al., 2021), and further investigation of the suggested cellular phenotype.

## Results

### Loss of Functional *DNAJC3* Alters Proteomic Signature of Human Fibroblasts

Three human primary fibroblast lines carrying previously reported pathogenic homozygous *DNAJC3* mutations (two with c.580C > T; p.Arg194^∗^ and one with a large deletion of 72 kb, spanning the *UGGT2* gene NM_006260.5) alongside three fibroblast lines of age-matched healthy controls were used for our proteomics analysis. While the large deletion is spanning the *UGGT2* gene, which can raise concerns of possible overlapping phenotypes, all DNAJC3 patients present with very similar phenotypes. No disease has been so far associated with mutations in the *UGGT2* gene.

**FIGURE 1:**
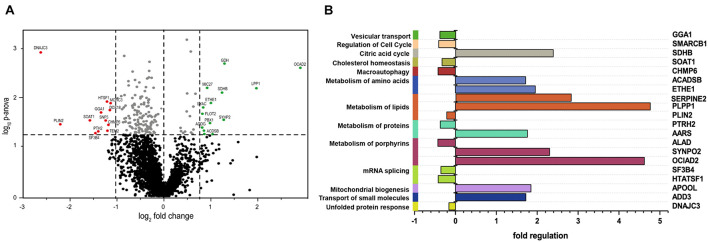
Comparative proteomic profiling results of *DNAJC3*-patient-derived fibroblasts versus control ones. **(A)** The volcano plot depicts all proteins identified in fibroblasts, making a clear delimitation between the statistically significant and non-significant quantified proteins (*p*-ANOVA < 0.05; horizontal line). Proteins with decreased abundances are represented in red, while the upregulated ones are highlighted in green color. **(B)** *In silico* analyses of proteomic findings included pathway analysis of the regulated proteins. All proteins with altered abundances were searched using KEGG, DAVID, and Reactome databases for protein pathway annotation. Vulnerable pathways (left *Y*-axis) were color-coded and referred to affected proteins (right *Y*-axis). Fold of regulation of the individual proteins is indicated on the *X*-axis. GGA1, ADP-ribosylation factor-binding protein GGA1; SMARCB1, SWI/SNF-related matrix-associated actin-dependent regulator of chromatin subfamily B member 1; SDHB, succinate dehydrogenase; SOAT1, Sterol *O*-acyltransferase 1; CHMP6, charged multivesicular body protein 6; ACADSB, short/branched chain specific acyl-CoA dehydrogenase; ETHE1, persulfide dioxygenase ETHE1; SERPINE2, Glia-derived nexin; PLPP1, phospholipid phosphatase 1; PLIN2, perilipin-2; PTRH2, peptidyl-tRNA hydrolase 2; AARS, alanine–tRNA ligase, cytoplasmic; ALAD, delta-aminolevulinic acid dehydratase; SYNPO2, synaptopodin-2; OCIAD2, OCIA domain-containing protein 2; SF3B4, splicing factor 3B subunit 4; HTATSF1, HIV tat-specific factor 1; APOOL, MICOS complex subunit MIC27; ADD3, gamma-adducin; DNAJC3, DnaJ homolog subfamily C member 3.

We detected 2,531 proteins in total with a minimum of two unique peptides, of which 165 presented with a *p*-ANOVA < 0.05, and from these, 24 were considered as altered in abundance (12 are upregulated and 12 are downregulated). The most significantly downregulated protein was DNAJC3, which was expressed at log_2_−2.66 in DNAJC3^*mut*^ fibroblasts compared to controls, thus confirming that the mutations lead to reduced protein level and demonstrating the robustness of our untargeted proteomic profiling approach. Thresholds were set to 2 standard deviations from the median in either increase or decrease to identify the most significantly altered proteins, which are highlighted in Figure 1A.

Pathways associated with dysregulated proteins (KEGG, Reactome, and DAVID libraries; Figure 1B) include lipid homeostasis, specifically cholesterol metabolism, which was perturbed in the DNAJC3^*mut*^ cells (Figure 1B). Furthermore, a dysregulation of ADP-ribosylation factor-binding protein GGA1, involved in the processing of APP as an amyloid precursor (von Arnim et al., 2006), was also observed. Apolipoprotein O (APOOL) was increased, which has important roles in mitochondrial lipids and prevention of mitochondrial lipotoxicity (Montero et al., 2010; Turkieh et al., 2014), as well as the Succinate dehydrogenase subunit B (SDHB), a major subunit of mitochondrial respiratory complex II. We therefore identified loss of DNAJC3 to be associated with a proteomic signature of dysregulation of the ER, lipid and cholesterol metabolism, dysregulation of mitochondrial cholesterol processing, and induction of respiratory complex expression.

### Loss of Functional *DNAJC3* Results in Cellular Lipid Increase

**FIGURE 2:**
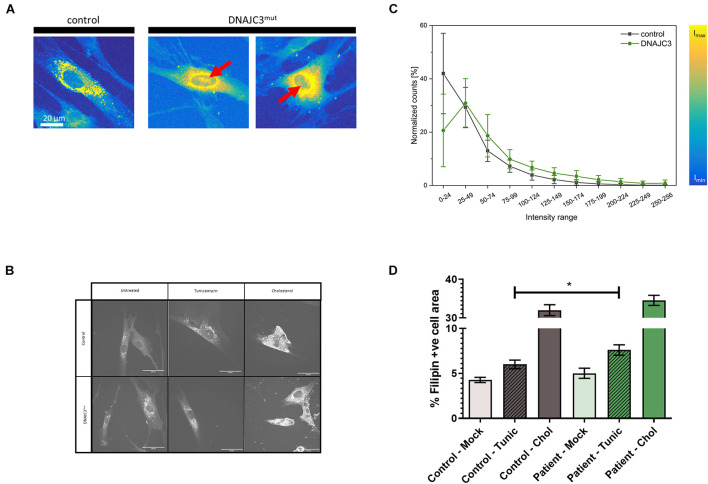
Lipid-level in DNAJC3-pathophysiology. **(A)** Results of BODIPY staining are shown for one representative fibroblast derived from controls (*left panel*) and two from DNAJC3 patients (*right panel*). Notably, in DNAJC3^*mut*^ cells, nuclear accumulated BODIPY staining can be observed (red arrows), which is absent in controls. **(B)** Single-cell histogram analysis: comparison of intensity histograms for DNAJC3^*mut*^ cells (green circles) with control cells (gray squares). Two samples per group were analyzed and at least 10 individual cells were used per sample for statistical analyses. For each cell, a histogram analysis was performed giving the frequency of intensity values in the specified ranges. Counts per intensity range were normalized to percentage values for individual cells before calculating the mean and standard deviation for each group. DNAJC3^*mut*^ fibroblasts tend to have more points with high fluorescence intensity than control cells, indicating an increased level of BODIPY stained lipids and thus an agglomeration/accumulation of lipids in these cells. **(C)** Confocal microscope images of Filipin III staining are shown for one representative fibroblast derived from controls and one DNAJC3 patient in the presence or absence of cholesterol or Tunicamycin. **(D)** Quantification of mean Filipin III positive area relative to cell area (%) for DNAJC3^*mut*^ and control fibroblasts. Both tunicamycin (Tunic) and cholesterol stress (Chol) treatments increase % Filipin positive area relative to control (*p* < 0.01, Student’s *t*-test with Welch’s correction) in both DNAJC3^*mut*^ and control, with * indicating statistical significance between DNAJC3^*mut*^ and control groups of the same treatment type.

Prompted by the proteomic findings and the fact that altered lipid homeostasis impacts a variety of cellular processes, such as energy homeostasis, signaling, and on a general note organelle homeostasis (Greenberg et al., 2011), we investigated perturbed lipid homeostasis in our control and DNAJC3^*mut*^ fibroblasts. Levels of triglycerides and cholesterol esters were investigated using the fluorescent neutral lipid dye 4,4-difluoro-1,3,5,7,8-pentamethyl-4-bora-3a,4a-diaza-s-indacene (BODIPY) and subsequent fluorescence microscopy. Results of these studies showed an increased diffuse staining with perinuclear accumulation and additional nuclear lipid-enrichment in DNAJC3^*mut*^ fibroblasts compared to the controls (Figures 2A,B), supporting the concept of altered lipid homeostasis in cells lacking functional DNAJC3.

As our proteomic data indicated a specific dysregulation of cholesterol metabolism, we studied free cholesterol levels using the filipin fluorescent staining technique (Figures 2C,D). While we did not observe an increase in free cholesterol under basal conditions, induction of ER stress with tunicamycin resulted in a significantly greater accumulation of cholesterol in DNAJC3^*mut*^ fibroblasts compared to controls, demonstrating that DNAJC3^*mut*^ fibroblasts have functional dysregulation of ER–cholesterol interactions. Treating cells with cholesterol and an inhibitor of Acyl-CoA-: cholesterol acyltransferase (ACAT-I) resulted in a significant increase of cholesterol in both controls and DNAJC3^*mut*^ fibroblasts (Figures 2C,D). This treatment saturated cholesterol clearance capacity and put both DNAJC3^*mut*^ and control fibroblasts under hypercholesterolemic stress, with only a non-significant slight increase observed in DNAJC3 patient cells compared to controls.

### The Endoplasmic Reticulum Is More Vulnerable Against Stress in DNAJC3^*mut*^ Fibroblasts

**FIGURE 3:**
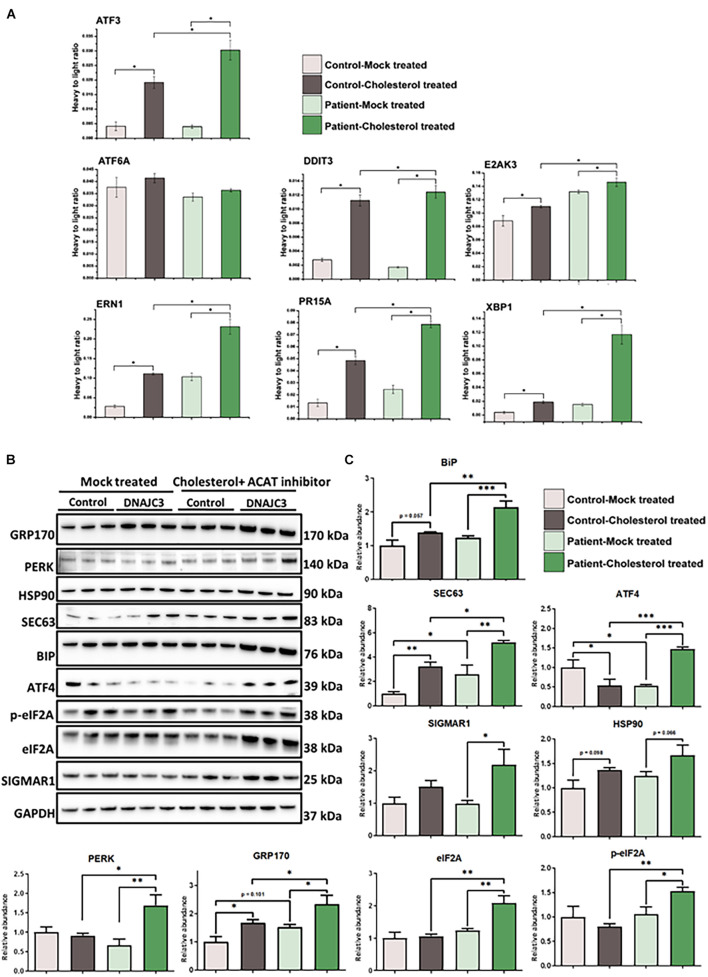
Endoplasmic reticulum (ER) stress in DNAJC3^*mut*^ fibroblasts. **(A)** Mean values (two technical and two biological replicates for each sample type, respectively) of PRM-based quantification of known ER stress-related proteins following treatment with either DMSO (mock) or soluble cholesterol + ACAT inhibitor in patient and control fibroblasts, respectively. Cholesterol treatment and concomitant inhibition of cholesterol acyltransferase (ACAT; esterifies cholesterol) resulted in pronounced ER stress in both DNAJC3^*mut*^ and control fibroblasts. Notably, significantly greater increases in ER stress protein expression was seen following soluble cholesterol treatment for ATF3 (*p* = 0.026), DDIT3 (*p* = 0.031), ERN1 (*p* = 0.049), PR15A (*p* = 0.028), and XBP1 (*p* = 0.003) in treated DNAJC3^*mut*^ cells. Student’s *t*-tests compared either mock-treated or cholesterol-treated control versus the correspondingly treated DNAJC3^*mut*^ fibroblasts. Student’s *t*-test (*n* = 2) of changes between mock-treated and cholesterol-treated expression levels is statistically significant between mock-treated DNAJC3^*mut*^ versus control fibroblasts for ATF3 (*p* = 0.027), DDIT3 (*p* = 0.031), ERN1 (*p* = 0.049), PR15A (*p* = 0.029), and XBP1 (*p* = 0.0033). Targeted peptides were ATF3: NFLIQQIK; ATF6: EAQDTSDGIIQK; DDIT3: VAQLAEENER; E2AK3: FLDNPHYNK; ERN1: FPNNLP; PR15A: GAALVEAGLEGEAR and XBP1: LLLENQLLR. **(B)** Results of immunoblot analysis of UPR markers in two DNAJC3-patient-derived and three control cells (treated under the same conditions as for the PRM ER stress investigations) showing changes in UPR markers in DNAJC3 cholesterol-treated cells, thus underlining the vulnerability of these cells to lipid accumulation. GAPDH was used to show equal loading. **(C)** Densitometry analysis of the immunoblotting from panel **(B)**. Graphs show mean±SD of triplicate samples (control fibroblasts and DNAJC3^*mut*^ fibroblasts under mock-treated and cholesterol-treated). For statistical analysis, Fisher’s LSD test was employed where ^∗^*p* ≤ 0.05, ^∗∗^*p* ≤ 0.01, ^∗∗∗^*p* ≤ 0.001 was considered as statistically significant.

It was previously shown that in the presence of tunicamycin or thapsigargin, which perturbs ER function and thus affects the secretory machinery, DNAJC3 mutant fibroblasts exhibit ER dysfunction (Synofzik et al., 2014). An increased sensitivity against additional ER stress burden was identified in DNAJC3-silenced rat and human β cells (Lytrivi et al., 2021). Notably, several publications demonstrated that cellular cholesterol accumulation leads to UPR activation (Feng et al., 2003; Cunha et al., 2008; Fu et al., 2011). DNAJC3^*mut*^ fibroblasts present a higher tendency of cholesterol aggregation compared to control cells (Figures 2C,D). Taking these findings into consideration, and the fact that neurons (as the vulnerable cellular population of the DNAJC3-phenotype) present with a more pronounced sensitivity against both ER stress and lipid accumulation (Jazvinscak Jembrek et al., 2015), DNAJC3^*mut*^ and control fibroblasts were further stressed by cholesterol overload to exacerbate ER stress. Parallel reaction monitoring (PRM) was employed to evaluate the respective activation of the UPR (Figure 3A). Statistically significant increases (*p* < 0.05) following cholesterol overload were observed for targets in both patient and control fibroblasts. Remarkably, ATF3, DDIT3, ERN1, PR15A, and XBP1 were significantly increased in DNAJC3^*mut*^ fibroblasts compared to controls (Figure 3A). To further elucidate an increased sensitivity of DNAJC3^*mut*^ fibroblasts against additional ER stress burden, additional immunoblot studies were carried out: BiP, major chaperone of the ER and a key modulator of UPR (Dudek et al., 2009), along with its co-chaperone GRP170 and SIGMAR1, SEC63, and HSP90. We observed an increase in BIP, SEC63, and SIGMAR1 proteins in DNAJC3^*mut*^ fibroblasts compared to controls. Furthermore, cholesterol treatment leads to a more significant increase of these proteins in DNAJC3^*mut*^ fibroblasts than controls, suggestive of an increased vulnerability toward ER stress upon cholesterol loading (Figures 3B,C). We observed a considerable increase in eIF2α phosphorylation, a master regulator of protein translation upon stress (Boye and Grallert, 2020), while PERK shows a mild increase in DNAJC3^*mut*^ fibroblasts after cholesterol-treatment (Figures 3B,C), which constitutes a hallmark of ER stress and UPR activation. ATF4 shows a decrease in cholesterol-stressed control fibroblasts in contrast with the increase observed in DNAJC3^*mut*^ cells after treatment (Figures 3B,C). Moreover, ATF6, one of the main UPR branches, seems unaffected by the loss of function of DNAJC3. Our combined proteomic and immunoblot findings suggest that loss of DNAJC3 leads to a very mild UPR activation with a slight increase of some UPR-modulated proteins under basal conditions, while further stress caused by cholesterol accumulation leads to an increase in UPR-associated proteins, which is more severe in DNAJC3^*mut*^ cells (Figure 3).

### Cholesterol Accumulation Affects Mitochondrial Homeostasis

**FIGURE 4:**
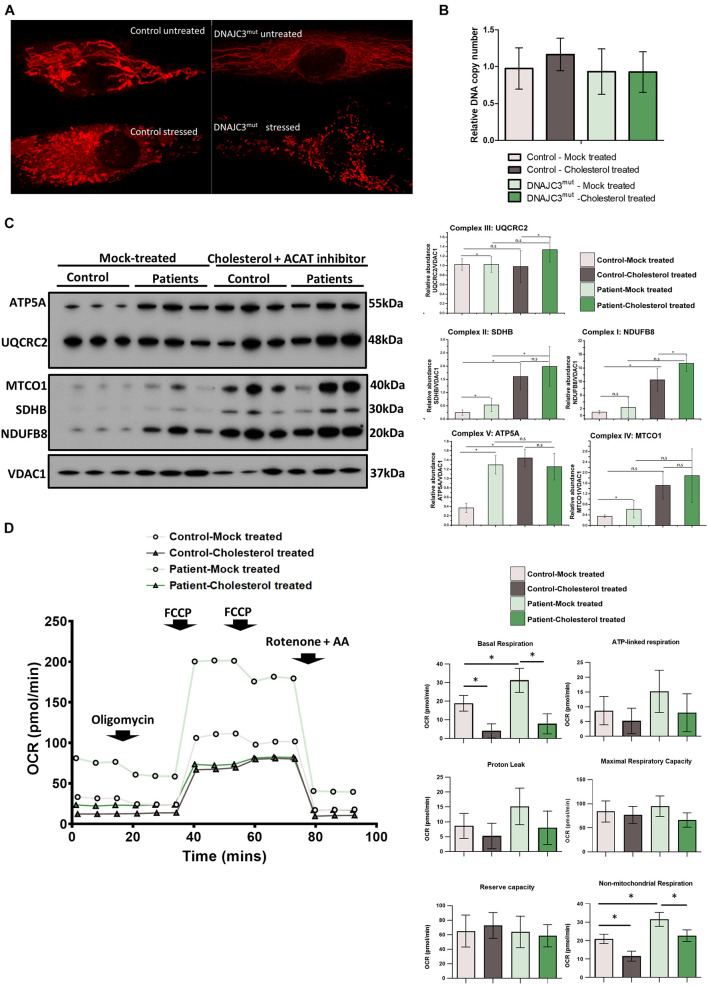
Study of mitochondrial integrity in DNAJC3 pathophysiology. **(A)** Mitotracker staining in DNAJC3^*mut*^ and control fibroblasts revealed a more fragmented mitochondria in patient cells with reduced staining in DNAJC3^*mut*^ cells compared to controls. One representative cell is presented per condition, whereby 30 cells have been studied for each of the analyzed conditions. **(B)** Mean mitochondrial DNA copy number quantitative real-time PCR relative abundances (two technical and two biological replicates for each sample type, respectively) show that cholesterol overload results in a mild increase of mtDNA copy in control but not in DNAJC3^*mut*^ fibroblasts. **(C)** Immunoblot studies of the mitochondrial respiratory chain complexes I–V (*left panel*) revealed a significant increase of complex I and II as well as IV and V in DNAJC3^*mut*^ cells, which was more pronounced in DNAJC3^*mut*^ cells after cholesterol-treatment; ^∗^*t*-test < 0.05. Level of the proteins representing the different complexes have been normalized to VDAC1. Densitometry analysis of the Western blotting results is represented in the right panel. **(D)** Mean oxygen consumption rate (OCR) of two biological replicates for each condition (each consisting of ≥12 technical replicates). Statistical significance indicated between treated versus non-treated DNAJC^*mut*^ or control cells, and between DNAJC^*mut*^ versus control cells under either treated or non-treated conditions. Student’s *t*-test, ^∗^*p* < 0.05.

The upregulation of several proteins involved in mitochondrial biogenesis observed in the DNAJC3^*mut*^ proteome (Figure 1) prompted us to examine the mitochondrial function in DNAJC3^*mut*^ fibroblasts. We evaluated mitochondrial function and the structure of mitochondrial networks in control and DNAJC3^*mut*^ fibroblasts using a redox staining (Mitotracker), with and without cholesterol treatment. Cholesterol treatment induced a clear fragmentation of the mitochondrial network in DNAJC3^*mut*^ and control cells (Figure 4A), indicating that cholesterol treatment induced combined ER/mitochondrial stress. As network imaging by redox staining is dependent on the membrane potential, differences in dye incorporation as a result of cholesterol treatment may be caused by changes in mitochondrial membrane potential. However, further studies would be needed to clarify this.

Next, we investigated mitochondrial copy number in control and DNAJC3^*mut*^ fibroblasts under basal conditions and after cholesterol treatment. Neither loss of DNAJC3 or cholesterol treatment affected mtDNA copy number, despite the associated changes in mitochondrial morphology (Figure 4B).

To further elucidate the effects upon loss of functional DNAJC3 on mitochondria, immunoblot studies focusing on expression of all five mitochondrial complexes were performed. DNAJC3^*mut*^ fibroblasts display a statistically significant increase in complex I (NDUFB8), complex II (SDHB), complex III (UQCRC2), and complex V (ATP5A) compared to controls, suggestive of a oxidative phosphorylation (OXPHOS) defect. Cholesterol treatment induces an increase in in mitochondrial complexes in both control and patient fibroblasts (Figure 4C) with a statistically significant increase of complexes I and III in DNAJC3 cells compared to controls (Figure 4C). These biochemical findings suggest that cholesterol accumulation leads to secondary upregulation of the respiratory complexes in DNAJC3^*mut*^ and, less prominently, in control cells (Figure 4C).

To determine whether the upregulation observed in the OXPHOS system has a functional effect on bioenergetic capacity, we performed oxygen consumption studies by Seahorse assay. DNAJC3^*mut*^ cells have increased basal respiration compared to controls, in accordance with the increased levels of mitochondrial complexes. In contrast, cholesterol-treated DNAJC3^*mut*^ and control cells show a decrease in basal respiration, ATP-linked respiration, and non-mitochondrial respiration compared to mock-treated cells. The decrease in maximal respiratory capacity after cholesterol treatment was greater in DNAJC3^*mut*^ cells compared to controls, suggesting that cholesterol treatment is compromising the compensatory capacity of DNAJC3^*mut*^ cells (Figure 4D). Additionally, we can observe an increase in non-mitochondrial respiration (Figure 4D) in DNAJC3^*mut*^ cells compared to controls, suggestive of the hindered bioenergetic health of the cells. This type of respiration has been suggested to increase in the presence of different stressors such as oxygen reactive species (Dranka et al., 2010; Hill et al., 2012).

### *DNAJC3* Mutant Fibroblasts Have Disrupted Regulation of Intracellular Amyloid Precursor Protein

**FIGURE 5:**
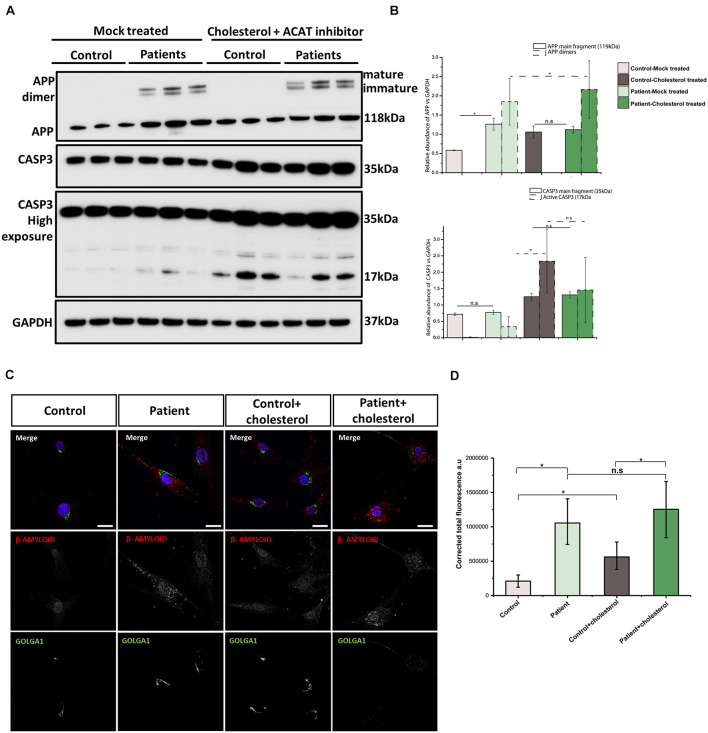
Analysis of amyloid accumulation in DNAJC3^*mut*^ cells. **(A)** Immunoblotting of APP reveals increased levels of this protein monomer in DNAJC3^*mut*^ cells, while cholesterol treatment leads to a further increase only in control cells. **(B)** Densitometry analysis of immunoblotting from panel **(A)**. Graphs show ±SD of triplicate samples, and all data were normalized to GAPDH. The mature and immature APP dimers on immunoblotting are present only in the DNAJC3^*mut*^ cells and cholesterol treatment increases the amount of these proteins, which are further cleaved to form the β-amyloid (Aβ) fragment. Cholesterol treatment induced an activation of caspase-3 protein (corresponding to the 17-kDa fragment), indicating an increase in apoptosis Student’s *t*-test, *p < 0.05. **(C)** Representative Aβ and GOLOGA1 staining in control and DNAJC3^*mut*^ cells in the presence and absence of cholesterol. Bar plots represent the corrected total florescence measured for the Aβ in three DNAJC3^*mut*^ and three controls in mock and cholesterol-treated cells. Immunofluorescence analysis shows that DNAJC3^*mut*^ fibroblasts present an increase in Aβ while the cholesterol treatment exacerbates the accumulation of the toxic fragment in patient cells. The cholesterol treatment leads to a slight increase in the Aβ in control cells as well; *t-test < 0.05.

ER stress may lead to an accumulation of β-amyloid (Aβ) and amyloid precursor protein (APP), and indeed stimulation of expression of other DnaJ family BiP co-chaperones has been shown to ameliorate the accumulation of Aβ in cellular Alzheimer’s disease models (Evans et al., 2006; Mansson et al., 2014). Moreover, impaired cholesterol homeostasis has been previously linked to Aβ accumulation (Ledesma and Dotti, 2006). These previously reported findings prompted us to study APP and Aβ in DNAJC3^*mut*^ fibroblasts compared to controls. Our findings show that cellular loss of functional DNAJC3 results in a significant increase of monomeric APP (Figure 5B). Moreover, DNAJC3^*mut*^ fibroblasts present two extra bands at higher molecular weight corresponding to the immature and mature dimeric form of APP (Figure 5B). Similar to DNAJC3^*mut*^ cells, cholesterol treatment in control cells leads to an increased level of APP compared to the mock-treated controls but without dimerization of APP (Figures 5A,B), suggesting that DNAJC3 loss impacts APP dimerization. The increase in the mature and immature APP dimers after cholesterol treatment in DNAJC3^*mut*^ cells suggests that the additional ER stress triggered by cholesterol treatment and cholesterol accumulation exacerbates this process. It is also worth noting that cholesterol treatment of DNAJC3^*mut*^ fibroblasts has no impact on the monomeric APP (Figures 5A,B).

Next, we assessed the apoptosis in our cell models. Both DNAJC3^*mut*^ and control cells show similar levels of procaspase (uncleaved caspase-3), while the cleavage product that indicate caspase-3 (CASP3) activation increases in DNAJC3^*mut*^ cells (Figures 5A,B). Cholesterol treatment led to increased CASP3 activation in both patient and control cells, suggesting that the increased stress burden of lipid accumulation induces apoptosis.

DNAJC3^*mut*^ cells display increased immunoreactivity to anti-Aβ antibody compared to control cells (Figure 5C). Cholesterol treatment leads to a significant increase in controls, while patient cells present a non-significant, smaller increase when compared to mock-treated DNAJC3 cells. Interestingly, despite cholesterol accumulation not significantly modifying the amount of Aβ deposition in DNAJC3 cells, we can observe that it induces a major change in the pattern of Aβ, which forms bigger clusters, found mostly around the perinuclear and nuclear region (Figure 5C). Cholesterol treatment of control cells does not seem to induce a change in Aβ cluster size and pattern as seen in treated mutant cells. Given that anti-Aβ staining does not detect Aβ in controls cultured under basal conditions, cholesterol treatment seems to trigger accumulation of Aβ even in controls. This finding in turn supports the concept that lipid buildup leads to an increase of Aβ peptide production (Shobab et al., 2005).

Intracellular trafficking of APP through Golgi is crucial for the generation of Aβ (Choy et al., 2012). Thus, we investigated Golgi integrity in fibroblasts using immunofluorescence (Figure 5C). Our data show a tightly compacted Golgi in perinuclear localization in control cells while some DNAJC3^*mut*^ cells present with a more sparce Golgi. Cholesterol treatment does not have any structural effects in controls, while DNAJC3^*mut*^ display a scattered Golgi, suggesting a vulnerability of this compartment upon lipid loading in patient cells.

## Discussion

Bi-allelic loss-of-function mutations in *DNAJC3*, encoding a co-chaperone of BiP, one of the major chaperones of the ER, have been shown to cause neurodegeneration with early-onset ataxia, upper-motor-neuron signs, demyelinating neuropathy, and cerebral atrophy associated with diabetes mellitus (Synofzik et al., 2014). Mutations in genes encoding BiP co-chaperones, localized in the ER or proteins involved in the UPR-modulation have been associated with central and peripheral nervous system abnormalities and non-autoimmune type insulin-dependent diabetes. For example, SIL1 is one of the main co-chaperones of BiP involved in the ATP-ADP cycle, and recessive *SIL1* mutations have been associated with MSS characterized by congenital cataracts, cerebellar ataxia, progressive muscle weakness, and delayed psychomotor development as well as axonal degeneration and disintegration of the neuromuscular junctions (Phan et al., 2019). Clinically overlapping features have been found in patients with recessive *DNAJC3* mutations (Synofzik et al., 2014). Remarkably, a modulating function of DNAJC3 expression in cerebellar degeneration has been demonstrated in the molecular etiology of MSS (Zhao et al., 2010), demonstrating a crucial role of this co-chaperone in neuronal maintenance. Other examples are Wolfram syndrome, caused by mutations in *WFS1* presenting with early-onset ataxia, cognitive deficits, and hearing loss, thus also including clinical hallmarks of the phenotype associated with *DNAJC3* mutations (Khanim et al., 2001) and Wollcot-Rallison syndrome, an autosomal recessive disorder caused by mutations in eukaryotic translation initiation factor 2-alpha kinase 3 (*E2AK3*), characterized by insulin-dependent diabetes, growth retardation, and intellectual disability (Delepine et al., 2000). In addition, a mutation in the *Sec61a1* gene (p.Tyr344His) results in excessive ER stress and apoptosis of pancreatic β cells in C57BL/6 mice, resulting in diabetes and hepatosteatosis (Linxweiler et al., 2017).

To obtain insights into the mechanism underlying the pathophysiology of mutations in *DNAJC3*, unbiased proteomic studies utilizing patient-derived fibroblasts were carried out (Figure 1). While fibroblasts are not the primary site of clinical manifestation, these cell models have been previously shown to be suitable to study the etiology of rare neurological diseases (Hentschel et al., 2021) and show very similar levels of DNAJC3 expression to peripheral nerve (Supplementary Figure 1). Results of this profiling suggest that lipid metabolism is affected by DNAJC3^*mut*^ mutations (Figure 1) as Sterol-*O*-acyltransferase 1 (SOAT1), an enzyme responsible for the esterification of fatty acids, especially cholesterol (Das et al., 2008), was found to be decreased. Lower levels of SOAT1 were previously linked to a hindered cholesterol clearance suggesting that DNAJC3-deficient cells present an accumulation of cholesterol (Tabas, 2002; Luo et al., 2010). We detected decreased abundance of Periplin 2 (PLIN2), an enzyme involved in lipid droplet formation, which has been linked to acute cholesterol accumulation (Makino et al., 2016), further suggesting a lipid dysregulation in fibroblasts lacking functional DNAJC3. Our functional data on cholesterol and lipid accumulation using immunofluorescence bring further evidence of lipid accumulation in DNAJC3^*mut*^ cells (Figure 2). Cholesterol homeostasis is essential for neuronal functioning and brain development, while perturbed cholesterol metabolism is a major pathophysiological mechanism in neurological diseases such as Alzheimer’s disease and Niemann-Pick type C disease, an autosomal recessive storage disorder, characterized by abnormal sequestration of unesterified cholesterol in cells (Fernandez et al., 2009; Marquer et al., 2014).

Label-free proteomics revealed that DNAJC3 is the most decreased protein in the mutant fibroblasts in alignment with previously published results (Synofzik et al., 2014), emphasizing the sensitivity of our proteomic profiling approach. Although DNAJC3 has been widely acknowledged in the attenuation of proteins involved in the initial ER response (Schorr et al., 2015), changes in ER morphology or increased ER calcium leakage were not described previously in the context of the underlying pathophysiology (Synofzik et al., 2014). However, an increased sensitivity against additional ER stress burden was identified in *DNAJC3*-silenced rat and human β cells (Lytrivi et al., 2021). In accordance with these previous observations, our findings suggest only a minor UPR-activation mirrored by the altered abundance of ER-stress-related proteins (Figure 3). DNAJC3 has been shown to bind the kinase domain of PERK (also called EIF2AK3), in order to terminate the first phase of ER stress (Yan et al., 2002). Given that DNAJC3^*mut*^ fibroblasts show slightly increased PERK expression (Figures 3B,C), its activation is presumably not terminated by DNAJC3 loss of function.

The ER has been described to tightly regulate the lipid metabolism as it is involved in the synthesis of phospholipids, re-esterification of sterols, and lipid-membrane biosynthesis (Volmer and Ron, 2015). Cholesterol accumulation disturbs the free cholesterol:phospholipid ratio on the normally fluid ER membrane, thus leading to the activation of ER stress (Feng et al., 2003). The impaired lipid/cholesterol metabolism in DNAJC3^*mut*^ cells was demonstrated by our Filipin-III and BODIPY staining affects ER maintenance and results in irregular lipid accumulation, supporting the concept of perturbed ER membrane structure. Additionally, induction of ER stress with tunicamycin leads to an increase in cholesterol accumulation in both control and patient cells, but with a more pronounced effect in DNAJC3 cells. We detected increased vulnerability of the ER in DNAJC3^*mut*^ cells after cholesterol treatment (Figure 3) similar to a previous study on *DNAJC3*-silenced rat and human β cells (Lytrivi et al., 2021). These findings provide evidence that lipid accumulation leads to ER stress activation, which is exacerbated in DNAJC3^*mut*^ patient cells.

There are numerous contact sites between ER and mitochondria [Mitochondria Associated ER Membranes (MAM)], which may enable cholesterol influx from the ER to the mitochondria (Arenas et al., 2017). The interaction of ER and mitochondrial membranes links mitochondrial dysfunction to ER stress. Our proteomic profiling data indicated that DNAJC3^*mut*^ fibroblasts present with mitochondrial alterations. We found that proteins involved in the maintenance of mitochondrial architecture and biogenesis (APOOL), fatty acid metabolism (ACDSB), or mitochondrial respiratory chain complexes (such as complex II, SDHB) are dysregulated as a results of DNAJC3 loss, suggestive of mitochondrial dysfunction. Further investigation in DNAJC3^*mut*^ fibroblasts revealed significantly increased level of mitochondrial respiratory chain subunits (except for complex IV) (Figure 4C) in line with the significant increase in basal respiration, which can be attributed to a possible increase in cellular energy demands. Mitochondrial respiration has been shown previously to increase due to ER stress (Balsa et al., 2019) and can constitute a pro-survival mechanism from ER stress (Knupp et al., 2019).

Mitochondria are organelles sparse in cholesterol; however, the transport of this lipid to the inner mitochondrial membrane *via* MAM proteins is tightly regulated and of physiological importance (Rone et al., 2009; Marriott et al., 2012). Cholesterol treatment of control and DNAJC3^*mut*^ fibroblast cells induced clear mitochondrial fragmentation altering the dynamics of the mitochondrial membranes (Figure 4) and leads to an increase in OXPHOS complexes in control cells, and an even greater increase in Complex I and III in DNAJC3^*mut*^ cells when compared to controls (Figure 4C). These changes have been previously associated with depletion of glutathione (GSH) (Fernandez et al., 2009). An increase of complex III has been associated with lower GSH levels and with an increase in mitochondrial depolarization, which leads to the release of Cytochrome *c* and activation of caspase-3 (Fernandez et al., 2009). Lipid overload increases caspase-3 levels in both control and patient cells, showing that cholesterol accumulation is a pro-apoptotic factor (Figures 5A,C). This may suggest that cholesterol overload exacerbates the DNAJC3^*mut*^ phenotype to further deplete GSH levels, finally leading to apoptosis. Further experiments focusing on apoptosis key factors would be needed to determine exactly the role of DNJC3 and lipid accumulation in cell death promotion. These findings accord with previous studies in DNAJC3-silenced rat and human β cells (Lytrivi et al., 2021), and in turn could also explain why non-mitochondrial respiration increases dramatically in DNAJC3^*mut*^ fibroblasts and in cells exposed to cholesterol (Figure 4D).

Endoplasmic reticulum stress, mitochondrial dysfunction, and cholesterol metabolism are thought to cause type 2 diabetes mellitus *via* activation of inflammatory pathways thereby inhibiting insulin secretion. Cholesterol treatment of pancreatic β cells triggers apoptosis and ER stress, while inhibition of cholesterol prevents β-cell apoptosis. However, *DNAJC3*-silencing rat and human β cells did not result in perturbed insulin production (Lytrivi et al., 2021).

β-Amyloid regulates the cholesterol flux to the mitochondria and interacts with components of the OXPHOS and thus participates in the molecular etiology of neurodegenerative diseases (Fernandez et al., 2009; Barbero-Camps et al., 2014). Disturbed cholesterol homeostasis has also been shown to affect Aβ accumulation, as cholesterol enhanced the aggregation of Aβ by inhibiting the degradation of this peptide (Yip et al., 2001; Ehehalt et al., 2003). Additionally, the C terminal APP fragment has been shown to regulate lipid homeostasis by acting as a cholesterol sensor in the membrane, contributing to the early pathology in Alzheimer’s disease (DelBove et al., 2019; Montesinos et al., 2020). Here, we detected the altered level of GGA1 (Figure 1), a Golgi protein that controls APP processing (von Arnim et al., 2006) and Golgi alterations (Figure 5C), suggesting that Aβ accumulation (a known hallmark in different neurodegenerative diseases) might be involved in cellular perturbations in DNAJC3^*mut*^cells. Indeed, DNAJC3^*mut*^ cells present with an increase in APP, which is not further affected by cholesterol treatment (Figure 5). We also detected two additional bands in the immunoblot corresponding to the mature and immature APP dimer (*N*- and *O*-glycosylated forms of APP dimers) (Isbert et al., 2012), supporting the concept of Aβ accumulation in DNAJC3^*mut*^ cells, which may further contribute to the neurodegenerative phenotype.

In summary, we show that *DNAJC3* mutations affect cellular lipid metabolism leading to (i) altered ER sensitivity toward stress burden, (ii) mitochondrial vulnerability, and (iii) Aβ accumulation. Neurons are particularly vulnerable to dysfunction of these pathways, which may explain the specifically neurological manifestation of DNAJC3 deficiency. Hence, our study provides molecular and mechanistic insights into the underlying pathophysiology of this rare disease and informs of the physiological function of DNAJC3.

## Materials and Methods

### Cell Culture

We studied two fibroblast lines carrying a homozygous c.580C > T (NM_006260.4, p.Arg194^∗^) premature stop mutation in *DNAJC3*, one line having a large homozygous deletion of 72 kb spanning *DNAJC3* (and the adjacent *UGGT2* gene), and control fibroblasts from three healthy donors, age and sex matched (Synofzik et al., 2014). Both mutations result in a 50% truncation of *DNAJC3* mRNA (Synofzik et al., 2014). Patient fibroblasts were cultured in Dulbecco’s Modified Eagle Media (DMEM) containing 4.5 g/L glucose, L-glutamine (Gibco) and phenol red supplemented with 10% fetal bovine serum (FBS) (Gibco) and 1% (v/v) penicillin/streptomycin solution at 37°C in a 5% CO_2_ atmosphere. Fibroblasts were grown to 80% confluence prior to harvesting or applying treatment.

### Cholesterol Stress Treatment

Fibroblasts (patient and controls) were stressed by supplementing the growth media with 10 μg/ml Sandoz 58-035 (Sigma-Aldrich) (solubilized in DMSO), an inhibitor of Acyl-CoA-: cholesterol acyltransferase (ACAT-I), for 6 h followed by further supplementation with 30 μg/ml of water-soluble cholesterol (Sigma-Aldrich). After 18 h of exposure to the Sandoz 58-035 and cholesterol, the stress treatment is considered complete; this is referred to now only as “Cholesterol-treated.” Patient and control fibroblasts were separately mock-treated with 0.1% DMSO and used as controls, referred to as “mock-treated.”

### Tunicamycin Treatment of Fibroblasts

Fibroblasts were treated for 18 h with 4 μg/ml Tunicamycin (Sigma-Aldrich). The stock solution of Tunicamycin was prepared using DMSO; thus, control and patient cells were treated with the same amount of DMSO as the Tunicamycin-treated cells and are referred to as “mock treated.”

### Label Free LC-MS/MS Analysis

#### Reagents

Ammonium hydrogen carbonate (NH_4_HCO_3_), anhydrous magnesium chloride (MgCl_2_), guanidine hydrochloride (GuHCl), iodoacetamide (IAA), and urea were purchased from Sigma-Aldrich, Steinheim, Germany. Tris base was obtained from Applichem Biochemica, Darmstadt, Germany and sodium dodecyl sulfate (SDS) was purchased from Carl Roth, Karlsruhe, Germany. Dithiothreitol (DTT) and EDTA-free protease inhibitor (Complete Mini) tablets were obtained from Roche Diagnostics, Mannheim, Germany. Sodium chloride (NaCl) and calcium chloride (CaCl_2_) were from Merck, Darmstadt. Sequencing grade modified trypsin was from Promega, Madison, WI USA. Benzonase^®^ Nuclease was purchased from Novagen. Bicinchoninic acid assay (BCA) kit was acquired from Thermo Fisher Scientific, Dreieich, Germany. All chemicals for ultra-pure HPLC solvents such as formic acid (FA), trifluoroacetic acid (TFA), and acetonitrile (ACN) were obtained from Biosolve, Valkenswaard, Netherlands.

#### Cell Lysis, Sample Clean-Up, and Proteolysis

Fibroblasts of patients with *DNAJC3* mutations (see above) and controls were processed independently. Cells were lysed in 100 μl of 50 mM Tris–HCl (pH 7.8) buffer containing 150 mM NaCl, 1% SDS, and EDTA-free protease inhibitor (Complete Mini) on ice for 15 min. Then samples were centrifuged for 5 min at 4°C and 6,000 × *g* and the protein concentration was determined by BCA assay (according to the manufacturer’s protocol). Cysteines were reduced by the addition of 10 mM DTT and samples were incubated at 56°C for 30 min, followed by alkylation of free thiol groups with 30 mM IAA at room temperature (RT) in the dark for 30 min.

Sample preparation was performed using filter-aided sample preparation (FASP) with some minor changes: 100 μg of protein lysate was diluted 10-fold with freshly prepared 8 M urea/100 mM Tris-HCl buffer (pH 8.5) (Burkhart et al., 2012; Kollipara and Zahedi, 2013), placed on PALL microsep centrifugal device (30 kDa cutoff), and centrifuged at 13,500 × *g* at RT for 20 min. Three washing steps were carried out with 100 μl of 8 M urea/100 mM Tris–HCl (pH 8.5) and then the buffer was exchanged by washing the device thrice with 100 μl of 50 mM NH_4_HCO_3_ (pH 7.8). One hundred microliters of digestion buffer [trypsin (Promega) (1:25 w/w, protease to substrate), 0.2 M GuHCl, and 2 mM CaCl_2_ in 50 mM NH_4_HCO_3_ (pH 7.8)] was added to the concentrated proteins, and the samples were incubated at 37°C for 14 h. Resulting tryptic peptides were recovered by centrifugation with 50 μl of 50 mM NH_4_HCO_3_ followed by 50 μl of ultra-pure water, and the resulting peptides were acidified [pH < 3 by addition of 10% TFA (v/v)]. All digests were quality controlled as described previously (Burkhart et al., 2012).

#### LC-MS/MS Measurement and Data Analysis

One microgram of each sample was measured using an Ultimate 3000 nano RSLC system coupled to an Orbitrap Fusion Lumos mass spectrometer (both Thermo Scientific) and analyzed in a randomized order to minimize systematic errors. Peptides were preconcentrated on a 100 μm × 2 cm C18 trapping column for 10 min using 0.1% TFA (v/v) at a flow rate of 20 μl/min followed by separation on a 75 μm × 50 cm C18 main column (both Pepmap, Thermo Scientific) with a 120-min LC gradient ranging from 3 to 35% of 84% ACN, 0.1% FA (v/v) at a flow rate of 250 nl/min. MS survey scans were acquired in the Orbitrap from 300 to 1,500 *m*/*z* at a resolution of 120,000 using the polysiloxane ion at *m*/*z* 445.12002 as lock mass (Olsen et al., 2005), an automatic gain control target value of 2.0 × 10^5^ and maximum injection times of 50 ms. Top 15 most intense signals were selected for fragmentation by HCD with a collision energy of 30% and MS/MS spectra were acquired in the Iontrap using an automatic gain control target value of 2.0 × 10^5^, a maximum injection time of 300 ms, a dynamic exclusion of 15 s.

Data analysis was performed using the Progenesis LC-MS software from Non-linear Dynamics (Newcastle upon Tyne, United Kingdom). Raw MS data were aligned by Progenesis, which automatically selected one of the LC-MS files as reference. After automatic peak picking, only features within retention time and *m/z* windows from 0 to 120 min and 300–1,500 *m/z*, with charge states +2, +3, and +4 were considered for peptide statistics and analysis of variance (ANOVA) and MS/MS spectra were exported as peak lists. Peak lists were searched against a concatenated target/decoy version of the human UniProt database (downloaded on July 22, 2015 containing 20,273 target sequences) using Mascot 2.4 (Matrix Science, Boston, MA, United States), MS-GF+, X!Tandem, and MyriMatch with the help of searchGUI 3.2.5 (Vaudel et al., 2011). Trypsin was selected as enzyme with a maximum of two missed cleavages, carbamidomethylation of Cys was set as fixed, and oxidation of Met was selected as variable modification. MS and MS/MS tolerances were set to 10 ppm and 0.5 Da, respectively.

To obtain peptide-spectrum match and to maximize the number of identified peptides and proteins at a given quality, we used PeptideShaker software 1.4.0 (Vaudel et al., 2015). Combined search results were filtered at a false discovery rate (FDR) of 1% on the peptide and protein level and exported using the PeptideShaker features that allow direct re-import of the quality-controlled data into Progenesis. Only proteins that were quantified with unique peptides were exported. For each protein, the average of the normalized abundances (obtained from Progenesis) from the analyses was calculated in order to determine the ratios between the patient and control fibroblast lysates. Only proteins that were (i) commonly quantified in all the replicates with (ii) one unique peptide, (iii) an ANOVA *p*-value of ≤0.05 (Progenesis), and (iv) an average log_2_ ratio ≤−1.17 or ≥1.7 were considered as dysregulated.

Pathway analysis on the label-free proteomics data was performed using GO term analysis (biological function) enrichment utilizing UniProt (available on www.uniprot.com).

### Parallel Reaction Monitoring of Unfolded Protein Response

The synthetic isotopic labeled (SIL) peptides were synthesized in-house and absolute quantified using amino acid analysis (AAA), which was performed as previously described (Nguyen et al., 2019). Tryptic peptides from cholesterol-treated and mock-treated DNAJC3^*mut*^ and control cells were obtained *via* FASP as described in the previous section. For the measurements, between 212.9 and 1,907.5 amol of the SIL peptides was spiked in each sample containing ∼1 μg of total tryptic peptides. The amounts and sequence of the spiked-in peptide are listed in Supplementary Table 1. For this experiment, we have employed two DNAJC3^*mut*^ patient cell lines from which one has a homozygous c.580C > T (NM_006260.4, p.Arg194^∗^) premature stop mutation in *DNAJC3* and one has a large homozygous deletion of 72 kb spanning *DNAJC3* and two age- and sex-matched controls.

#### Liquid Chromatography and Mass Spectrometry

For the separation and detection of peptides, an Ultimate 3000 Rapid Separation Liquid Chromatography (RSLC) nano system equipped with ProFlow flow control device was coupled to an Orbitrap Fusion Lumos Tribrid Mass Spectrometer equipped with a nano-electrospray ion source (all from Thermo Scientific, Bremen, Germany). The peptide separation was performed as described previously (Nguyen et al., 2019). The nano-electrospray ion source was run using positive ion mode at a spray voltage of 1,800 V. The mass detection and quantification were performed with the normalized collision energy at 32%, automated gain control (AGC) target value at 2 × 10^5^, resolution at 240,000, isolation width at a mass-to-charge value of 0.4, and injection time at 600 ms.

#### Data Analysis

Skyline 64-bit version 3.7.0.11317 (MacLean et al., 2010) was used to generate methods and analyze the raw data after measurement. All generated data were reviewed and integrated manually. The choice of peptide as well as the monitored fragments were adapted from Nguyen et al. (2019). The light-to-heavy ratio was built from the total area of all monitored fragments of endogenous peptides (light) and SIL peptides (heavy). All the measurements were performed with two technical replicates.

### Mitochondrial Network Analysis

Mitotracker-redTM (MTR) (ThermoFisher), a membrane potential-sensitive dye, was used to label the mitochondria in fibroblasts. Cells were seeded to glass-bottomed dishes 24 h prior to cholesterol stress treatment. Fibroblast growth media was aspirated, and cells were incubated in staining media constituted of minimal essential medium (Gibco), without phenol-red, 10% FBS, and 75 nM MTR for 30 min at 37°C prior to imaging.

Imaging of the mitochondrial network was performed using a Nikon A1R confocal microscope to capture three-dimensional stacks of images, which were then processed to make two-dimensional maximum intensity projections of the mitochondrial network.

### Mitochondrial Oxygen Consumption Assay

For this experiment, we use two DNAJC3^*mut*^ patient cell lines, one with homozygous c.580C > T (NM_006260.4, p.Arg194^∗^) premature stop mutation in *DNAJC3* and the other a large homozygous deletion of 72 kb spanning *DNAJC3*, with two age- and sex-matched healthy controls. The oxygen consumption rate (OCR) of fibroblasts was measured using an XF96 Extracellular Flux Analyzer (Seahorse Bioscience, Agilent Technologies). Fibroblasts were seeded into wells of the 96-well cell culture microplate (Seahorse Bioscience) in 80 μl for 24 h prior to cholesterol stress treatment in the microplate. After this, cell media was replaced with 180 μl of bicarbonate-free DMEM and incubated for 30 min. Supplementation of the media with oligomycin, carbonyl cyanide-*p*-trifluoromethoxy-phenylhydrazone (FCCP), rotenone, and antimycin allows the determination of oxygen consumption rates attributable to basal respiration, proton leakage, maximal respiratory capacity, and non-mitochondrial respiration. ATP-linked respiration and reserve capacity were also calculated. Inhibition of ATP synthase by supplementation of media with oligomycin allows the calculation of ATP-synthesis-linked oxygen consumption. Subsequent supplementation with the uncoupling agent maximizes the respiratory rate. OCR levels were normalized to cellular mass, estimated by total well protein measured by Bradford assay.

### Quantification of Mitochondrial DNA Copy Number

The relative mtDNA copy number per cell was quantified by a multiplex Taqman (Bio-rad 4369510) qPCR assay by amplifying *MT-ND1* (mitochondrial encoded gene) and *B2M* (nuclear encoded gene) with a CFX96^TM^ Real-Time PCR Detection System (Bio-Rad) following the protocol described previously (Bartsakoulia et al., 2016). The primers used for template generation of standard curves and the qPCR reaction are as follows B2M: Fw-CACTGAAAAAGATGAGTATGCC, Rv-AA CATTCCCTGACAATCCC; MTND1: Fw-AACA TTCCCTGA CAATCCC, Rv-AACATTCCCTGACAATCCC. Copies per microliter of each template were standardized to 1 × 10^10^ and a 10-fold dilution series was amplified, with a DNA negative control on each plate. This was performed in 20 μl reactions in a 96-well plate (Bio-Rad 5496), sealed using microplate “B” plate sealers (VWR 391-1293). The reaction mixture was composed of: 5 μl × 5 × Taqman (Bio-rad 4369510), 0.4 μM of reverse and forward primers, 25–50 ng of DNA template, 0.2 μl of MyTaq HS DNA polymerase (Bioline BIO-21112), and PCR-grade autoclaved sterile deionized water (to make up 20 μl of reaction mixture). The cycling conditions were as follows: (1) initial denaturation at 95°C for 3 min, (2) 40 cycles of denaturation at 95°C for 10 s, and (3) annealing and extension at 62.5°C for 1 min. The relative mtDNA copy number was calculated using the ΔCt data following the equation: CopyNumber = 2 (2^–Δ*Ct*^) where Delta Ct (ΔCt) equals the sample Ct of the mitochondrial gene (MTND1) subtracted from the sample Ct of the nuclear reference gene (B2M).

### Immunoblot Analyses

Whole protein extracts of cholesterol- and mock-treated fibroblasts were prepared by lysing cells with RIPA buffer (Sigma Aldrich) containing a protease inhibitor cocktail (Roche). Proteins were separated by utilizing Bis-Tris Gels (Invitrogen) and transferred to PVDF membranes (Invitrogen). Membranes were blocked for 2 h at room temperature with 5% milk in PBS-T and incubated with primary antibodies at 4°C overnight. The primary antibodies and dilutions used are as follows: mouse anti-APP A4 1:1,000 (Merck-Millipore), mouse anti-GRP78 1:2,000 (BD Biosciences), rabbit anti-CASP3 1:1,000 (AB clonal), mouse anti-GAPDH 1:2,000 (Abcam), mouse anti-VDAC1 1:1,000 (Abcam), and mouse anti-total OXPHOS antibody cocktail 1:1,000 (Abcam). Protein signals were detected with a Super SignalTM West Pico PLUS (Thermo Scientific) kit according to the manufacturer’s protocol.

### Immunofluorescence Studies

For imaging amyloid protein distribution and the Golgi network, cells were grown on coverslips and then fixed with 4% formaldehyde for 15 min at room temperature. Excessive formaldehyde was removed, and samples washed thrice with PBS before permeabilization with 0.5% Triton X in PBS for 10 min at room temperature and then blocked with 1% BSA for 1 h at room temperature. Next, cells were washed thrice with 1 ml of PBS and then incubated at 4°C overnight with an anti-Golgin-97 (rabbit polyclonal, GeneTex) and anti-beta-amyloid (mouse monoclonal, DE2B4, Abcam) primary antibodies. Excessive primary antibody was removed by washing three times with 1 ml of PBS. Fluorescently labeled antibodies rabbit Alexa Fluor 488 and mouse Alexa Fluor 594 (both Thermo Fisher Scientific) diluted in 1% BSA in PBS were added to the samples and incubated at room temperature for 1 h. Samples were washed finally three times with PBS and once very briefly with water and mounted onto microscope slides using ProLong Gold Antifade Mountant with DAPI (Thermo Fisher Scientific). Imaging was performed using a Nikon A1R confocal microscope and acquired images were further analyzed using the open source image-processing package in Fiji.

### Cellular Cholesterol and Lipid Quantification Using Florescence Microscopy

To visualize lipid loading in our cells, we have employed Filipin and BODIPY staining. Filipin III was employed to specifically visualize cholesterol deposits in our cell models.

Cell culture medium was removed, and cells were gently washed thrice with 1 × PBS (room temperature). Next, 100 μl of 4% formaldehyde was added to each coverslip and incubated for 15 min at room-temperature. After the formaldehyde has been removed, cells were again washed thrice with 1 × PBS (room-temperature). Cells were next incubated with 1 ml of 1.5 mg/ml glycine at room temperature for 10 min. Filipin III from *Streptomyces filipinensis* (Sigma-Aldrich F4767) was added on cells (125 μg/ml) for 2 h at room temperature. Cells were next rinsed three times with PBS and slides were mounted using ProLong Gold Antifade Mountant (Thermo Fisher Scientific). Imaging was performed using a Leica confocal microscope (excitation approximately 360 nm and emission approximately 480 nm).

Data analysis was performed using Fiji. Two thresholds were set; the higher one to include only filipin-positive areas, and the lower one to also include areas of cellular autofluorescence as a measure of total cellular area. From this percentage, filipin positivity relative to cellular area was calculated for each image, and the mean was determined for all images of the fibroblast/treatment combination to give the percentage filipin positivity per combination. A minimum of 55 cells per combination were employed for quantification.

BODIPY (493/503; green solution; Life technologies, catalog # D-3922) was added followed by an incubation overnight at 4°C in the dark. The next day, BODIPY was removed, and cells were washed three times with 200 μl of PBS and stored at 4°C until microscopic inspection was carried out. Fluorescence measurements were performed on a modified Leica TCS SP8 CARS laser scanning microscope using a 25× water immersion objective [Fluotar VISIR 25x/0.95 WATER, for full description of the system see (Ebersbach et al., 2018)]. BODIPY fluorescence was excited at 488 nm and detected at 495–600 nm with a hybrid detector. Specifications of the hybrid detectors are given by the manufacturer. DAPI imaging was performed using 405 nm for excitation and detection of the fluorescence at 415–475 nm with a PMT. Both fluorescence measurements were carried out sequentially for each sample position. Multiple cell images were acquired as 3D stacks with a resolution of 2,048 × 2,048 pixels and a step size of 227 nm in *x* and *y* direction and five to nine layers in the *z* direction with a step size of 570 nm. 3D images of single cells were acquired with the same objective and a resolution of 512 × 512 pixels using a step size of 303 nm in the *x* and *y* direction and 570 nm in the *z* direction.

All data processing was performed using Matlab R2015a. For comparability of the different samples, all data were preprocessed as follows: For each 3D measurement, the mean intensity was calculated in the *z* direction. For the resulting 2D image, background noise was reduced by setting all data points with less than 1% of intensity to this lower threshold. To account for cosmic spikes, an upper intensity threshold was set so that less than 0.1% of the data points showed fluorescence intensity above this value. Intensities exceeding this upper limit were set to this value. Images were then rescaled to full range (8-bit) between these two threshold values.

For statistical analyses, single-cell images were cut from the mean images using an irregular octagon. For each of these cells, an intensity histogram analysis was performed. To account for the different sizes of the single-cell images, the histogram values were normalized to percentage values. From the analyzed cells per sample, the mean and standard deviation were calculated on the normalized histogram data.

### Plotting and Statistical Analysis

Data were plotted using GraphPad Prism v.7.0 software (GraphPad Software, United States) or Origin 6.0 (Origin Lab) and Adobe Illustrator Artwork 23.0 (Adobe Systems). The statistical test and method are indicated in the legend of the figures. *p*-values of less than 0.05 were considered statistically significant for all experiments.

## Data Availability Statement

The datasets presented in this study can be found in online repositories. The names of the repository/repositories and accession number(s) can be found below: ProteomeXchange with identifier PXD028168 and PXD027372.

## Ethics Statement

The studies involving human participants were reviewed and approved by Ethics Committee of University of Medicine Essen, Germany (19-9011-BO).

## Author Contributions

DH and AR completed the label free proteomics studies while CN and RA performed the targeted UPR measurements. DH and MJJ performed western blotting of UPR components, filipin staining, and mitochondrial analysis. UM and EF performed the BODIPY staining and analysis. BM studied mtDNA copy numbers. AS, IE, and MS contributed the constructive comments and aided in data interpretation. MS contributed also with the fibroblasts utilized for this study. MJJ, DH, RH, and AR were involved in the design of the experiments, data analysis, and wrote the manuscript. All authors contributed to the article and approved the submitted version.

## Conflict of Interest

The authors declare that the research was conducted in the absence of any commercial or financial relationships that could be construed as a potential conflict of interest.

## Publisher’s Note

All claims expressed in this article are solely those of the authors and do not necessarily represent those of their affiliated organizations, or those of the publisher, the editors and the reviewers. Any product that may be evaluated in this article, or claim that may be made by its manufacturer, is not guaranteed or endorsed by the publisher.

## Supplementary Material

Supplementary Figure 1 DNAJC3 expression in different tissues (brain, skin, and muscle) as depicted on GTEx Portal (https://www.gtexportal.org/home/).

## Abbreviations

ER: endoplasmic reticulum
DNAJC3: DnaJ heat shock protein family (Hsp40) member C3
UPR: unfolded protein response
PERK: protein kinase R (PKR)-like endoplasmic reticulum kinase
eIF2- α: eukaryotic initiation factor 2A
A β: β-amyloid
mtDNA: mitochondrial DNA
MSS: Marinesco–Sjörgen syndrome
ATP/ADP: adenosine tri/di-phosphate.

## References

[B1] Anttonen A. K. Mahjneh I. Hamalainen R. H. Lagier-Tourenne C. Kopra O. Waris L. (2005). The gene disrupted in Marinesco-Sjogren syndrome encodes SIL1, an HSPA5 cochaperone. *Nat. Genet.* 37 1309–1311. 10.1038/ng1677 16282978

[B2] Arenas F. Garcia-Ruiz C. Fernandez-Checa J. C. (2017). Intracellular cholesterol trafficking and impact in neurodegeneration. *Front. Mol. Neurosci.* 10:382. 10.3389/fnmol.2017.00382 29204109 PMC5698305

[B3] Balsa E. Soustek M. S. Thomas A. Cogliati S. Garcia-Poyatos C. Martin-Garcia E. (2019). ER and nutrient stress promote assembly of respiratory chain supercomplexes through the PERK-eIF2alpha axis. *Mol. Cell* 74 877–890.e6. 10.1016/j.molcel.2019.03.031 31023583 PMC6555668

[B4] Barbero-Camps E. Fernandez A. Baulies A. Martinez L. Fernandez-Checa J. C. Colell A. (2014). Endoplasmic reticulum stress mediates amyloid beta neurotoxicity via mitochondrial cholesterol trafficking. *Am. J. Pathol.* 184 2066–2081. 10.1016/j.ajpath.2014.03.014 24815354 PMC4076561

[B5] Bartsakoulia M. Mupsilonller J. S. Gomez-Duran A. Yu-Wai-Man P. Boczonadi V. Horvath R. (2016). Cysteine supplementation may be beneficial in a subgroup of mitochondrial translation deficiencies. *J. Neuromuscul. Dis.* 3 363–379. 10.3233/JND-160178 27854233

[B6] Blumen S. C. Astord S. Robin V. Vignaud L. Toumi N. Cieslik A. (2012). A rare recessive distal hereditary motor neuropathy with HSJ1 chaperone mutation. *Ann. Neurol.* 71 509–519. 10.1002/ana.22684 22522442

[B7] Boriushkin E. Wang J. J. Li J. Jing G. Seigel G. M. Zhang S. X. (2015). Identification of p58IPK as a novel neuroprotective factor for retinal neurons. *Invest. Ophthalmol. Vis. Sci.* 56 1374–1386. 10.1167/iovs.14-15196 25655802 PMC4340432

[B8] Boye E. Grallert B. (2020). eIF2alpha phosphorylation and the regulation of translation. *Curr. Genet.* 66 293–297. 10.1007/s00294-019-01026-1 31485739

[B9] Bublitz S. K. Alhaddad B. Synofzik M. Kuhl V. Lindner A. Freiberg C. (2017). Expanding the phenotype of DNAJC3 mutations: a case with hypothyroidism additionally to diabetes mellitus and multisystemic neurodegeneration. *Clin. Genet.* 92 561–562. 10.1111/cge.13069 28940199

[B10] Buchkremer S. Gonzalez Coraspe J. A. Weis J. Roos A. (2016). Sil1-mutant mice elucidate chaperone function in neurological disorders. *J. Neuromuscul. Dis.* 3 169–181. 10.3233/JND-160152 27854219 PMC5271578

[B11] Burkhart J. M. Schumbrutzki C. Wortelkamp S. Sickmann A. Zahedi R. P. (2012). Systematic and quantitative comparison of digest efficiency and specificity reveals the impact of trypsin quality on MS-based proteomics. *J. Proteomics* 75 1454–1462. 10.1016/j.jprot.2011.11.016 22166745

[B12] Choy R. W. Cheng Z. Schekman R. (2012). Amyloid precursor protein (APP) traffics from the cell surface via endosomes for amyloid beta (Abeta) production in the trans-Golgi network. *Proc. Natl. Acad. Sci. U.S.A.* 109 E2077–E2082. 10.1073/pnas.1208635109 22711829 PMC3409748

[B13] Cunha D. A. Hekerman P. Ladriere L. Bazarra-Castro A. Ortis F. Wakeham M. C. (2008). Initiation and execution of lipotoxic ER stress in pancreatic beta-cells. *J. Cell Sci.* 121 2308–2318. 10.1242/jcs.026062 18559892 PMC3675788

[B14] Das A. Davis M. A. Rudel L. L. (2008). Identification of putative active site residues of ACAT enzymes. *J. Lipid Res.* 49 1770–1781. 10.1194/jlr.M800131-JLR200 18480028 PMC2444009

[B15] DelBove C. E. Strothman C. E. Lazarenko R. M. Huang H. Sanders C. R. Zhang Q. (2019). Reciprocal modulation between amyloid precursor protein and synaptic membrane cholesterol revealed by live cell imaging. *Neurobiol. Dis.* 127 449–461. 10.1016/j.nbd.2019.03.009 30885793 PMC6588454

[B16] Delepine M. Nicolino M. Barrett T. Golamaully M. Lathrop G. M. Julier C. (2000). EIF2AK3, encoding translation initiation factor 2-alpha kinase 3, is mutated in patients with Wolcott-Rallison syndrome. *Nat. Genet.* 25 406–409. 10.1038/78085 10932183

[B17] Dranka B. P. Hill B. G. Darley-Usmar V. M. (2010). Mitochondrial reserve capacity in endothelial cells: the impact of nitric oxide and reactive oxygen species. *Free Radic. Biol. Med.* 48 905–914. 10.1016/j.freeradbiomed.2010.01.015 20093177 PMC2860730

[B18] Dudek J. Benedix J. Cappel S. Greiner M. Jalal C. Muller L. (2009). Functions and pathologies of BiP and its interaction partners. *Cell. Mol. Life Sci.* 66 1556–1569. 10.1007/s00018-009-8745-y 19151922 PMC11131517

[B19] Ebersbach P. Stehle F. Kayser O. Freier E. (2018). Chemical fingerprinting of single glandular trichomes of *Cannabis sativa* by Coherent anti-Stokes Raman scattering (CARS) microscopy. *BMC Plant Biol.* 18:275. 10.1186/s12870-018-1481-4 30419820 PMC6233497

[B20] Ehehalt R. Keller P. Haass C. Thiele C. Simons K. (2003). Amyloidogenic processing of the Alzheimer beta-amyloid precursor protein depends on lipid rafts. *J. Cell Biol.* 160 113–123. 10.1083/jcb.200207113 12515826 PMC2172747

[B21] Evans C. G. Wisen S. Gestwicki J. E. (2006). Heat shock proteins 70 and 90 inhibit early stages of amyloid beta-(1-42) aggregation *in vitro*. *J. Biol. Chem.* 281 33182–33191. 10.1074/jbc.M606192200 16973602

[B22] Feng B. Yao P. M. Li Y. Devlin C. M. Zhang D. Harding H. P. (2003). The endoplasmic reticulum is the site of cholesterol-induced cytotoxicity in macrophages. *Nat. Cell Biol.* 5 781–792. 10.1038/ncb1035 12907943

[B23] Fernandez A. Llacuna L. Fernandez-Checa J. C. Colell A. (2009). Mitochondrial cholesterol loading exacerbates amyloid beta peptide-induced inflammation and neurotoxicity. *J. Neurosci.* 29 6394–6405. 10.1523/JNEUROSCI.4909-08.2009 19458211 PMC2740839

[B24] Fu S. Yang L. Li P. Hofmann O. Dicker L. Hide W. (2011). Aberrant lipid metabolism disrupts calcium homeostasis causing liver endoplasmic reticulum stress in obesity. *Nature* 473 528–531. 10.1038/nature09968 21532591 PMC3102791

[B25] Greenberg A. S. Coleman R. A. Kraemer F. B. McManaman J. L. Obin M. S. Puri V. (2011). The role of lipid droplets in metabolic disease in rodents and humans. *J. Clin. Invest.* 121 2102–2110. 10.1172/JCI46069 21633178 PMC3104768

[B26] Hentschel A. Czech A. Munchberg U. Freier E. Schara-Schmidt U. Sickmann A. (2021). Protein signature of human skin fibroblasts allows the study of the molecular etiology of rare neurological diseases. *Orphanet. J. Rare Dis.* 16:73. 10.1186/s13023-020-01669-1 33563298 PMC7874489

[B27] Hill B. G. Benavides G. A. Lancaster J. R. Jr. Ballinger S. Dell’Italia L. Jianhua Z. (2012). Integration of cellular bioenergetics with mitochondrial quality control and autophagy. *Biol. Chem.* 393 1485–1512. 10.1515/hsz-2012-0198 23092819 PMC3594552

[B28] Ichhaporia V. P. Sanford T. Howes J. Marion T. N. Hendershot L. M. (2015). Sil1, a nucleotide exchange factor for BiP, is not required for antibody assembly or secretion. *Mol. Biol. Cell* 26 420–429. 10.1091/mbc.E14-09-1392 25473114 PMC4310734

[B29] Isbert S. Wagner K. Eggert S. Schweitzer A. Multhaup G. Weggen S. (2012). formation is initiated in the endoplasmic reticulum and differs between APP isoforms. *Cell. Mol. Life Sci.* 69 1353–1375. 10.1007/s00018-011-0882-4 22105709 PMC3314181

[B30] Jazvinscak Jembrek M. Hof P. R. Simic G. (2015). Ceramides in Alzheimer’s disease: key mediators of neuronal apoptosis induced by oxidative stress and abeta accumulation. *Oxid. Med. Cell. Longev.* 2015:346783. 10.1155/2015/346783 26090071 PMC4458271

[B31] Jin H. Mimura N. Kashio M. Koseki H. Aoe T. (2014). Late-onset of spinal neurodegeneration in knock-in mice expressing a mutant BiP. *PLoS One* 9:e112837. 10.1371/journal.pone.0112837 25405877 PMC4236098

[B32] Khanim F. Kirk J. Latif F. Barrett T. G. (2001). WFS1/Wolframin mutations, Wolfram syndrome, and associated diseases. *Hum. Mutat.* 17 357–367. 10.1002/humu.1110 11317350

[B33] Knupp J. Arvan P. Chang A. (2019). Increased mitochondrial respiration promotes survival from endoplasmic reticulum stress. *Cell Death Differ.* 26 487–501. 10.1038/s41418-018-0133-4 29795335 PMC6370866

[B34] Kollipara L. Zahedi R. P. (2013). Protein carbamylation: *in vivo* modification or *in vitro* artefact? *Proteomics* 13 941–944. 10.1002/pmic.201200452 23335428

[B35] Kollipara L. Buchkremer S. Coraspe J. A. G. Hathazi D. Senderek J. Weis J. (2017). In-depth phenotyping of lymphoblastoid cells suggests selective cellular vulnerability in Marinesco-Sjogren syndrome. *Oncotarget* 8 68493–68516. 10.18632/oncotarget.19663 28978133 PMC5620273

[B36] Krieger M. Roos A. Stendel C. Claeys K. G. Sonmez F. M. Baudis M. (2013). SIL1 mutations and clinical spectrum in patients with Marinesco-Sjogren syndrome. *Brain* 136 3634–3644. 10.1093/brain/awt283 24176978

[B37] Kulanuwat S. Tangjittipokin W. Jungtrakoon P. Chanprasert C. Sujjitjoon J. Binnima N. (2018). DNAJC3 mutation in Thai familial type 2 diabetes mellitus. *Int. J. Mol. Med.* 42 1064–1073. 10.3892/ijmm.2018.3678 29767246

[B38] Ledesma M. D. Dotti C. G. (2006). Amyloid excess in Alzheimer’s disease: what is cholesterol to be blamed for? *FEBS Lett.* 580 5525–5532. 10.1016/j.febslet.2006.06.038 16814780

[B39] Linxweiler M. Schick B. Zimmermann R. (2017). Let’s talk about Secs: Sec61, Sec62 and Sec63 in signal transduction, oncology and personalized medicine. *Signal Transduct. Target. Ther.* 2:17002. 10.1038/sigtrans.2017.2 29263911 PMC5661625

[B40] Luo D. X. Cao D. L. Xiong Y. Peng X. H. Liao D. F. (2010). A novel model of cholesterol efflux from lipid-loaded cells. *Acta Pharmacol. Sin.* 31 1243–1257. 10.1038/aps.2010.93 20835267 PMC4012915

[B41] Luo S. Mao C. Lee B. Lee A. S. (2006). GRP78/BiP is required for cell proliferation and protecting the inner cell mass from apoptosis during early mouse embryonic development. *Mol. Cell. Biol.* 26 5688–5697. 16847323 10.1128/MCB.00779-06 PMC1592753

[B42] Lytrivi M. Senee V. Salpea P. Fantuzzi F. Philippi A. Abdulkarim B. (2021). DNAJC3 deficiency induces beta-cell mitochondrial apoptosis and causes syndromic young-onset diabetes. *Eur. J. Endocrinol.* 184 459–472. 10.1530/EJE-20-0636 33486469

[B43] MacLean B. Tomazela D. M. Shulman N. Chambers M. Finney G. L. Frewen B. (2010). Skyline: an open source document editor for creating and analyzing targeted proteomics experiments. *Bioinformatics* 26 966–968. 20147306 10.1093/bioinformatics/btq054 PMC2844992

[B44] Makino A. Hullin-Matsuda F. Murate M. Abe M. Tomishige N. Fukuda M. (2016). Acute accumulation of free cholesterol induces the degradation of perilipin 2 and Rab18-dependent fusion of ER and lipid droplets in cultured human hepatocytes. *Mol. Biol. Cell* 27 3293–3304. 27582390 10.1091/mbc.E15-10-0730 PMC5170862

[B45] Mansson C. Arosio P. Hussein R. Kampinga H. H. Hashem R. M. Boelens W. C. (2014). Interaction of the molecular chaperone DNAJB6 with growing amyloid-beta 42 (Abeta42) aggregates leads to sub-stoichiometric inhibition of amyloid formation. *J. Biol. Chem.* 289 31066–31076. 25217638 10.1074/jbc.M114.595124 PMC4223311

[B46] Marquer C. Laine J. Dauphinot L. Hanbouch L. Lemercier-Neuillet C. Pierrot N. (2014). Increasing membrane cholesterol of neurons in culture recapitulates Alzheimer;s disease early phenotypes. *Mol. Neurodegener.* 9:60. 10.1186/1750-1326-9-60 PMC4280040 25524049

[B47] Marriott K. S. C. Prasad M. Thapliyal V. Bose H. S. (2012). sigma-1 receptor at the mitochondrial-associated endoplasmic reticulum membrane is responsible for mitochondrial metabolic regulation. *J. Pharmacol. Exp. Ther.* 343 578–586. 22923735 10.1124/jpet.112.198168 PMC3500540

[B48] Montero J. Mari M. Colell A. Morales A. Basañez G. Garcia-Ruiz C. (2010). Cholesterol and peroxidized cardiolipin in mitochondrial membrane properties, permeabilization and cell death. *Biochim. Biophys. Acta* 1797 1217–1224. 20153716 10.1016/j.bbabio.2010.02.010 PMC2889134

[B49] Montesinos J. Pera M. Larrea D. Guardia-Laguarta C. Agrawal R. R. Velasco K. R. (2020). The Alzheimer’s disease-associated C99 fragment of APP regulates cellular cholesterol trafficking. *EMBO J.* 39:e103791. 10.15252/embj.2019103791 PMC7560219 32865299

[B50] Nguyen C. D. L. Malchow S. Reich S. Steltgens S. Shuvaev K. V. Loroch S. (2019). A sensitive and simple targeted proteomics approach to quantify transcription factor and membrane proteins of the unfolded protein response pathway in glioblastoma cells. *Sci. Rep.* 9:8836. 10.1038/s41598-019-45237-5 PMC6586633 31222112

[B51] Olsen J. V. de Godoy L. M. Li G. Macek B. Mortensen P. Pesch R. (2005). Parts per million mass accuracy on an Orbitrap mass spectrometer via lock mass injection into a C-trap. *Mol. Cell Proteomics* 4 2010–2021. 16249172 10.1074/mcp.T500030-MCP200

[B52] Oyadomari S. Yun C. Fisher E. A. Kreglinger N. Kreibich G. Oyadomari M. (2006). Cotranslocational degradation protects the stressed endoplasmic reticulum from protein overload. *Cell* 126 727–739. 16923392 10.1016/j.cell.2006.06.051

[B53] Phan V. Cox D. Cipriani S. Spendiff S. Buchkremer S. O’Connor E. (2019). SIL1 deficiency causes degenerative changes of peripheral nerves and neuromuscular junctions in fish, mice and human. *Neurobiol. Dis.* 124 218–229. 30468864 10.1016/j.nbd.2018.11.019

[B54] Rone M. B. Fan J. J. Papadopoulos V. (2009). Cholesterol transport in steroid biosynthesis: role of protein-protein interactions and implications in disease states. *BBA Mol. Cell Biol.* 1791 646–658. 10.1016/j.bbalip.2009.03.001 19286473 PMC2757135

[B55] Roos A. Buchkremer S. Kollipara L. Labisch T. Gatz C. Zitzelsberger M. (2014). Myopathy in Marinesco-Sjogren syndrome links endoplasmic reticulum chaperone dysfunction to nuclear envelope pathology. *Acta Neuropathol.* 127 761–777. 10.1007/s00401-013-1224-4 24362440

[B56] Roos A. Kollipara L. Buchkremer S. Labisch T. Brauers E. Gatz C. (2016). Cellular signature of SIL1 depletion: disease pathogenesis due to alterations in protein composition beyond the ER machinery. *Mol. Neurobiol.* 53 5527–5541. 10.1007/s12035-015-9456-z 26468156

[B57] Rutkowski D. T. Kang S. W. Goodman A. G. Garrison J. L. Taunton J. Katze M. G. (2007). The role of p58IPK in protecting the stressed endoplasmic reticulum. *Mol. Biol. Cell* 18 3681–3691. 10.1091/mbc.e07-03-0272 17567950 PMC1951758

[B58] Schorr S. Klein M. C. Gamayun I. Melnyk A. Jung M. Schauble N. (2015). Co-chaperone specificity in gating of the polypeptide conducting channel in the membrane of the human endoplasmic reticulum. *J. Biol. Chem.* 290 18621–18635. 10.1074/jbc.M115.636639 26085089 PMC4513120

[B59] Senderek J. Krieger M. Stendel C. Bergmann C. Moser M. Breitbach-Faller N. (2005). Mutations in SIL1 cause Marinesco-Sjogren syndrome, a cerebellar ataxia with cataract and myopathy. *Nat. Genet.* 37 1312–1314. 16282977 10.1038/ng1678

[B60] Shobab L. A. Hsiung G. Y. R. Feldman H. H. (2005). Cholesterol in Alzheimer’s disease. *Lancet Neurol.* 4 841–852. 16297842 10.1016/S1474-4422(05)70248-9

[B61] Synofzik M. Haack T. B. Kopajtich R. Gorza M. Rapaport D. Greiner M. (2014). Absence of BiP co-chaperone DNAJC3 causes diabetes mellitus and multisystemic neurodegeneration. *Am. J. Hum. Genet.* 95 689–697. 25466870 10.1016/j.ajhg.2014.10.013 PMC4259973

[B62] Tabas I. (2002). Consequences of cellular cholesterol accumulation: basic concepts and physiological implications. *J. Clin. Invest.* 110 905–911. 10.1172/JCI0216452 12370266 PMC151158

[B63] Turkieh A. Caubère C. Barutaut M. Desmoulin F. Harmancey R. Galinier M. (2014). Apolipoprotein O is mitochondrial and promotes lipotoxicity in heart. *J. Clin. Invest.* 124 2277–2286. 10.1172/JCI74668 24743151 PMC4001558

[B64] Vaudel M. Barsnes H. Berven F. S. Sickmann A. Martens L. (2011). SearchGUI: an open-source graphical user interface for simultaneous OMSSA and X!Tandem searches. *Proteomics* 11 996–999. 10.1002/pmic.201000595 21337703

[B65] Vaudel M. Burkhart J. M. Zahedi R. P. Oveland E. Berven F. S. Sickmann A. (2015). PeptideShaker enables reanalysis of MS-derived proteomics data sets. *Nat. Biotechnol.* 33 22–24. 10.1038/nbt.3109 25574629

[B66] Volmer R. Ron D. (2015). Lipid-dependent regulation of the unfolded protein response. *Curr. Opin. Cell Biol.* 33 67–73. 10.1016/j.ceb.2014.12.002 25543896 PMC4376399

[B67] von Arnim C. A. F. Spoelgen R. Peltan I. D. Deng M. Courchesne S. Koker M. (2006). GGA1 acts as a spatial switch altering amyloid precursor protein trafficking and processing. *J. Neurosci.* 26 9913–9922. 10.1523/JNEUROSCI.2290-06.2006 17005855 PMC6674476

[B68] Yan W. Frank C. L. Korth M. J. Sopher B. L. Novoa I. Ron D. (2002). Control of PERK eIF2alpha kinase activity by the endoplasmic reticulum stress-induced molecular chaperone P58IPK. *Proc. Natl. Acad. Sci. U.S.A.* 99 15920–15925. 10.1073/pnas.252341799 12446838 PMC138540

[B69] Yip C. M. Elton E. A. Darabie A. A. Morrison M. R. McLaurin J. (2001). Cholesterol, a modulator of membrane-associated A beta-fibrillogenesis and neurotoxicity. *J. Mol. Biol.* 311 723–734. 10.1006/jmbi.2001.4881 11518526

[B70] Zhao L. Rosales C. Seburn K. Ron D. Ackerman S. L. (2010). Alteration of the unfolded protein response modifies neurodegeneration in a mouse model of Marinesco-Sjogren syndrome. *Hum. Mol. Genet.* 19 25–35. 10.1093/hmg/ddp464 19801575 PMC2792147

