# Supplementary figures and images for: Intracellular Lipid Accumulation and Mitochondrial Dysfunction Accompanies Endoplasmic Reticulum Stress Caused by Loss of the Co-chaperone DNAJC3
Source: Front Cell Dev Biol. 2021 Oct 6;9:710247. doi: 10.3389/fcell.2021.710247 (PMC8526738; doi:10.3389/fcell.2021.710247)

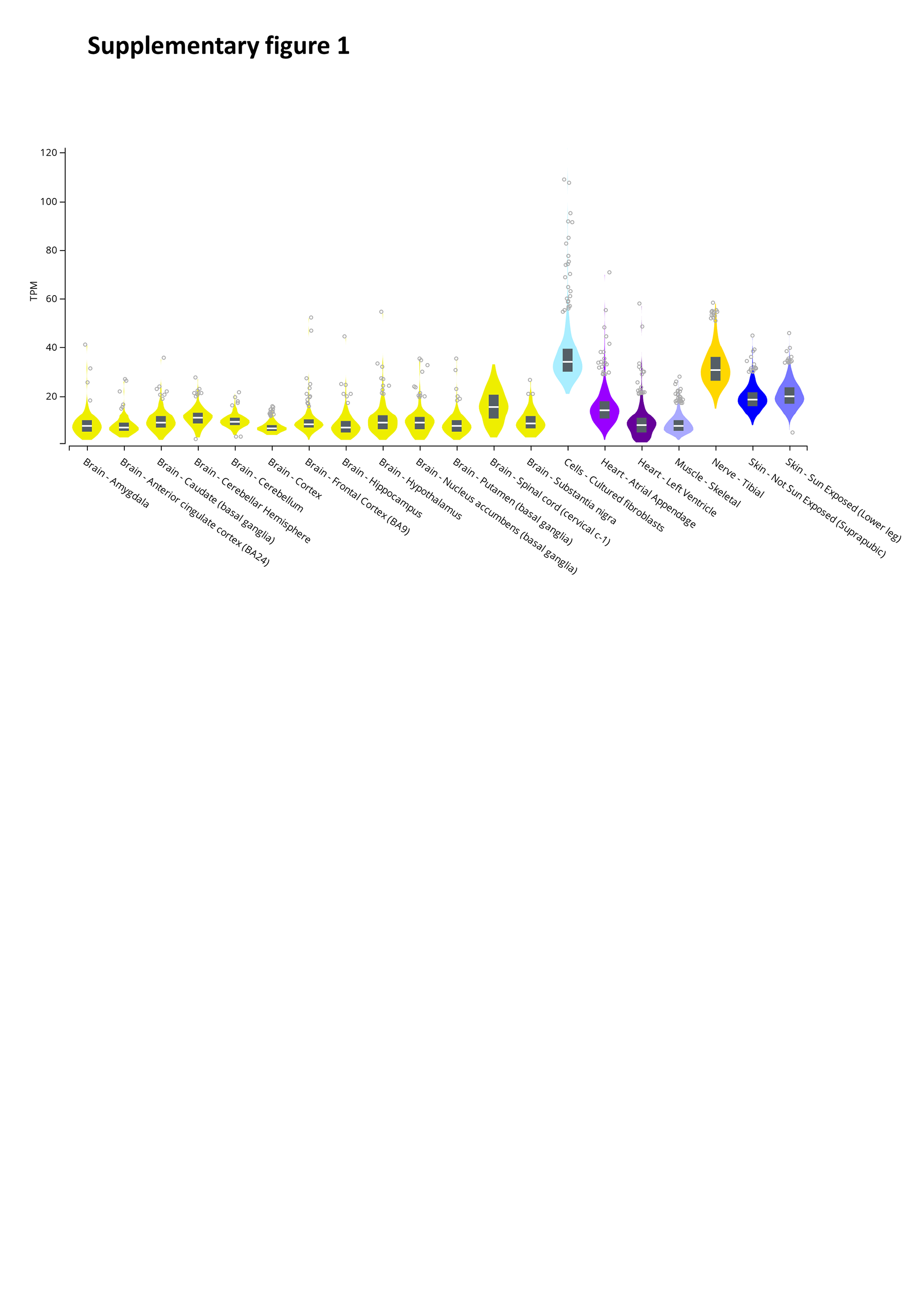

Supplement: Supplementary Figure 1 — DNAJC3 expression in different tissues (brain, skin, and muscle) as depicted on GTEx Portal (https://www.gtexportal.org/home/). [file Image_1.TIF]
